# Case Report: Neuroendocrine-low POU2F3-positive small cell lung cancer presenting as a diagnostically unstable thoracic malignancy

**DOI:** 10.3389/fonc.2026.1917451

**Published:** 2026-09-04

**Authors:** Ying Ma, Wenlong Chen, Fangfei Guo, Songchen Han

**Affiliations:** 1 Department of Radiology, Gansu Provincial Hospital, Lanzhou, China; 2 Gansu University of Chinese Medicine, Lanzhou, China; 3 Department of Cardiac Surgery, Gansu Provincial Hospital, Lanzhou, China; 4 Department of Thoracic Surgery, Gansu Provincial Hospital, Lanzhou, China; 5 Jinan University, Guangzhou, China

**Keywords:** diagnostic instability, minimal residual disease, multidisciplinary team, neuroendocrine-low, pathologic complete response, POU2F3, small cell lung cancer

## Abstract

POU2F3-positive small cell lung cancer (SCLC) is a tuft-cell-like, neuroendocrine-low subtype that may be difficult to classify in small specimens. We report a 61-year-old man with a left-upper-lobe high-grade thoracic malignancy radiographically staged as cT2aN2bM0. The cN2b component was imaging-based, and inflammatory nodal uptake could not be fully excluded because of previous pulmonary tuberculosis. Bronchoscopic biopsy from the opening of the left-upper-lobe lingular bronchus and CT-guided core biopsy of the primary mass sampled anatomically distinct sites within the same tumor process. Both samples showed morphology favoring small cell carcinoma, but conventional neuroendocrine markers were absent, focal, or weak and the immunophenotype varied by specimen and review. Morphology and specimen-specific immunophenotyping, integrated with specialist consultation, supported small cell carcinoma; POU2F3 nuclear positivity supported subtype assignment and explained the neuroendocrine-low phenotype rather than establishing the diagnosis alone. Nab-paclitaxel, cisplatin, and pembrolizumab were used as an empiric bridge strategy while histologic classification remained unresolved. After four induction cycles and marked radiographic response, a highly selected multidisciplinary decision led to surgery for pathologic assessment. Resection showed pCR/ypT0N0M0, followed by four postoperative cycles of TP plus pembrolizumab. On May 26, 2026, surveillance imaging showed no recurrence or metastasis; no ctDNA was detected on subsequent plasma testing, which was interpreted as MRD-negative. This single case is illustrative and does not define a treatment standard, a POU2F3-specific response phenotype, or evidence of cure

## Introduction

Small cell lung cancer (SCLC) is biologically heterogeneous, with transcription-factor-associated states that include ASCL1-, NEUROD1-, and POU2F3-associated tumors (1, 2). POU2F3-positive SCLC is commonly described as tuft-cell-like and neuroendocrine-low. Conventional neuroendocrine markers may be absent, focal, or weak, creating a diagnostic interface with other high-grade thoracic malignancies (3–6).

In small biopsies, morphology, anatomic sampling context, immunohistochemistry, molecular findings, and expert review must be interpreted together. TTF-1 negativity, variable neuroendocrine-marker expression, focal squamous-marker staining in an earlier batch, and retained RB1 can reduce confidence in a conventional diagnostic pathway. POU2F3 may help recognize a subtype, but it is not a stand-alone diagnostic marker for SCLC (4–6).

**Figure 1:**
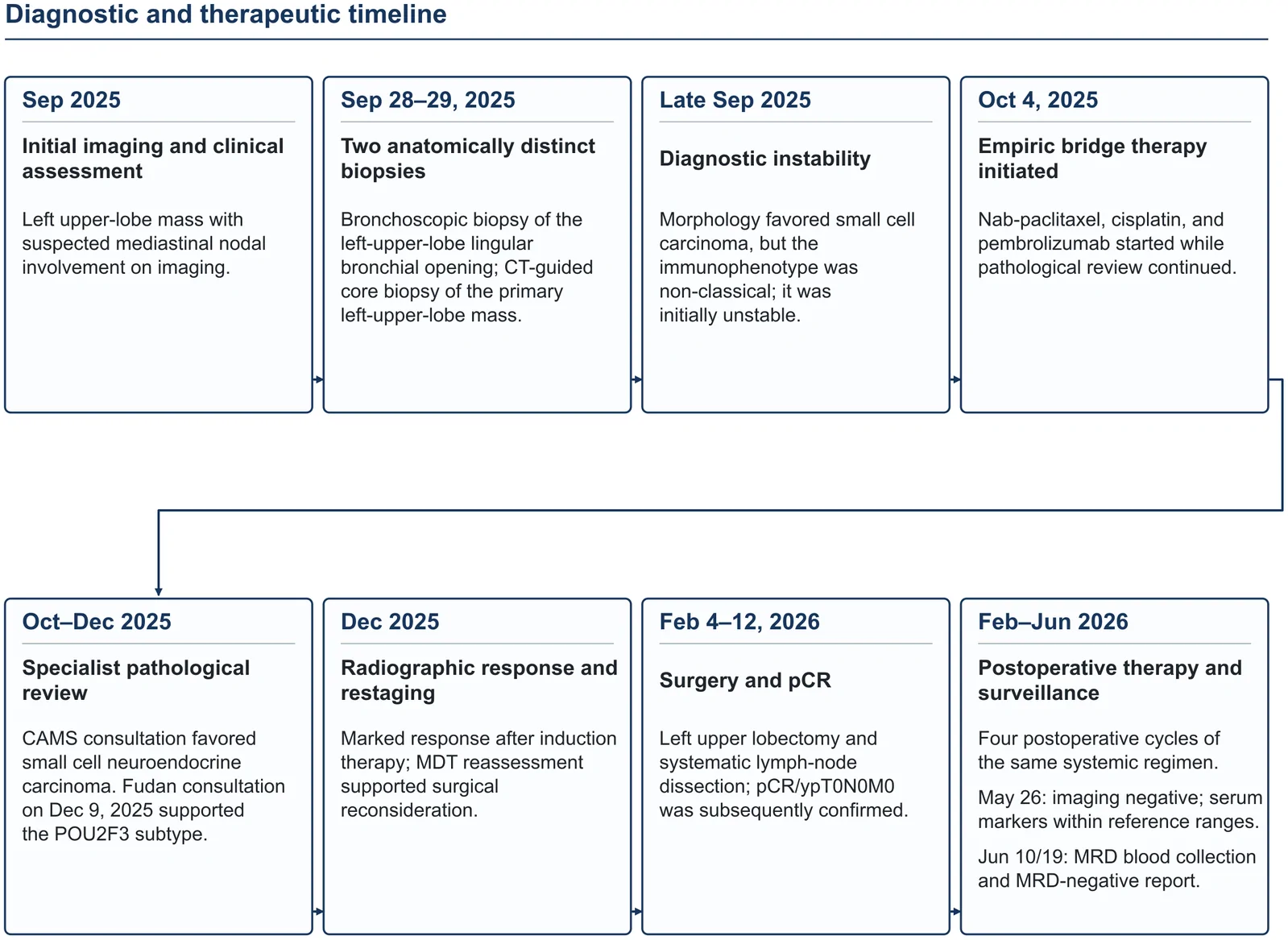
Chronological clinical timeline under diagnostic uncertainty. The timeline shows initial imaging and clinical assessment, followed by bronchoscopic biopsy from the opening of the left-upper-lobe lingular bronchus on September 28, 2025 and an anatomically distinct CT-guided core biopsy of the primary left-upper-lobe mass on September 29, 2025. Diagnostic instability was recognized, and empiric bridge therapy was initiated on October 4, 2025 while pathology review continued. Specialist consultations and POU2F3-supported subtype refinement followed. Response assessment and restaging occurred in December 2025, followed by surgery on February 4, 2026 and pCR/ypT0N0M0 confirmation on February 12, 2026. The subsequent course included four postoperative cycles of TP plus pembrolizumab, negative imaging and clinical follow-up on May 26, 2026, MRD blood collection on June 10, 2026, and an MRD-negative report on June 19, 2026. The sequence is individualized and does not define a standard treatment pathway.

This report follows CARE principles (7). The completed CARE checklist is available as **Supplementary Material**. It focuses on how an anatomically distinct two-site biopsy strategy, staged multidisciplinary reassessment, and postoperative pathology were used to manage diagnostic instability while avoiding therapeutic generalization from an individual outcome. The chronological diagnostic and therapeutic course is summarized in **Figure 1**.

## Case presentation

A 61-year-old man with grade 2 hypertension, previous pulmonary tuberculosis, a maternal history of lung cancer, and more than 30 years of smoking presented with intermittent cough and sputum production for more than 1 month. Serum tumor markers on September 30, 2025 showed normal ProGRP (33.30 pg/mL; reference <63.7) and NSE (10.96 ng/mL; reference <20), with mild elevations of SCC antigen (1.90 ng/mL; reference <1.5), CEA (6.29 ng/mL; reference <5), and CA19-9 (39.12 U/mL; reference <37). This profile was concordant with limited neuroendocrine-marker expression but was not used to assign either diagnosis or subtype.

PET/CT on September 28, 2025 showed an irregular left-upper-lobe lesion near the mediastinum, approximately 32 × 16 × 15 mm (SUVmax 12.49), and FDG-avid lymph nodes in stations 5 and 6 and the left hilum (SUVmax 8.57). No definite extrathoracic hypermetabolic metastasis was identified. The clinical stage was radiographic cT2aN2bM0. The cN2b designation was not pathologically proven because invasive nodal staging was not performed before induction therapy; prior tuberculosis also meant that inflammatory uptake in PET-positive nodes could not be fully excluded.

### Integrated pathology review across two anatomically distinct sampling sites

Bronchoscopic biopsy on September 28, 2025 sampled tumor involving the bronchial mucosa and native glands at the opening of the lingular bronchus of the left upper lobe. CT-guided core biopsy on September 29, 2025 sampled the primary left-upper-lobe mass. These were two anatomically distinct sampling sites within the same thoracic tumor process. In both samples, the reviewed tumor showed relatively small cells, scant cytoplasm, a high nuclear-to-cytoplasmic ratio, nuclear molding, finely granular chromatin, inconspicuous nucleoli, brisk mitotic activity, prominent apoptosis, necrosis, and crush artifact; the morphology favored small cell neuroendocrine carcinoma.

At Chinese Academy of Medical Sciences Cancer Hospital consultation, the bronchoscopic specimen showed CK7 3+ and was negative for TTF-1, Napsin A, chromogranin A, synaptophysin, INSM1, p40, p63, and CK5/6. CD117 was 2+, BRG1/SMARCA4 and RB1 were retained, p53 showed approximately 90% strong positivity with a missense-type overexpression pattern, and Ki-67 was approximately 40%.

An earlier originating-hospital batch had recorded focal or partial p40, p63, and CK5/6 positivity. These institution- and batch-specific results are reported separately rather than merged.

The CT-guided core showed CKpan 2+, CK7 2+, CK8/18 1+, and CD56 2 +. Synaptophysin was focal and weakly positive, and occasional INSM1-positive cells were present. Chromogranin A and NSE were negative. TTF-1, Napsin A, p40, p63, and CK5/6 were negative. CD117 was 3+, BRG1/SMARCA4 and INI1 were retained, NUT was negative, RB1 was retained, p53 showed approximately 95% strong positivity with a missense-type pattern, Ki-67 was approximately 90%, and PD-L1 SP263 TPS was approximately 1%.

The consultation conclusion was poorly differentiated neuroendocrine carcinoma favoring small cell carcinoma with low neuroendocrine-marker expression. A minor LCNEC component could not be completely excluded because occasional cells showed visible nucleoli and relatively more cytoplasm.

Fudan University Shanghai Cancer Center reviewed the two specimens separately and diagnosed each as poorly differentiated neuroendocrine carcinoma (small cell carcinoma, POU2F3 subtype). Both specimens showed POU2F3 nuclear positivity and CD56 positivity, with negative GATA3 and androgen receptor and Ki-67 approximately 90% or greater. The supplied report did not provide POU2F3 percentage, intensity, or focal-versus-diffuse distribution. Original slides were re-reviewed during specialist consultation; no new pretreatment experiment was performed for this revision.

Morphology and integrated pathology established the diagnosis of small cell carcinoma. POU2F3 supported subtype assignment and explained the neuroendocrine-low phenotype; it was not used as a stand-alone marker to establish SCLC. ASCL1, NEUROD1, and YAP1 were not performed, and pretreatment tissue was exhausted. NUT had already been tested in the CT-guided core and was negative.

Tissue-based sequencing of the CT-guided core biopsy identified TP53 p.Y236C with a variant allele frequency of 61.7%. Immunohistochemistry demonstrated a p53 overexpression pattern in both pretreatment specimens. These findings supported an aberrant TP53 pathway and a high-grade malignant phenotype but were not lineage-specific evidence for SCLC. The specimen-specific immunohistochemical and molecular findings across pretreatment biopsies and external consultations are summarized in **Table 1**.

**Table 1:** Specimen-specific immunohistochemical and molecular findings across pretreatment biopsies and external consultations.

Marker/feature	Bronchoscopic biopsy—originating hospital/CAMS	CT-guided core biopsy—originating hospital/CAMS	Fudan review—bronchoscopic specimen	Fudan review—CT-guided core	Interpretive note
Sampling site	Opening of the left-upper-lobe lingular bronchus	Primary left-upper-lobe mass	Opening of the left-upper-lobe lingular bronchus	Primary left-upper-lobe mass	Two anatomically distinct sites within the same thoracic tumor process
Morphology	Small cells; scant cytoplasm; high N:C ratio; molding; fine chromatin; mitoses, apoptosis, necrosis, and crush artifact	Same overall small-cell features; occasional cells with visible nucleoli and more cytoplasm	Poorly differentiated neuroendocrine carcinoma (small cell carcinoma, POU2F3 subtype)	Poorly differentiated neuroendocrine carcinoma (small cell carcinoma, POU2F3 subtype)	Both sites morphologically favored small cell carcinoma; minor LCNEC component not completely excluded in the core
CKpan/CK7/CK8/18	CKpan not listed; CK7 3+	CKpan 2+; CK7 2+; CK8/18 1+	Not reported in supplied summary	Not reported in supplied summary	Epithelial differentiation
CD56	not reported	2+	positive	positive	Expression varied by specimen and review.
Chromogranin A	negative	negative	not reported	not reported	Absent conventional neuroendocrine marker.
Synaptophysin	negative	focal weak positive	not reported	not reported	Limited conventional neuroendocrine marker expression.
NSE IHC	not reported	negative	not reported	not reported	Distinct from serum NSE.
INSM1	negative	occasional positive cells	not reported	not reported	Negative or limited expression.
TTF-1/Napsin A	negative/negative	negative/negative	not reported	not reported	No stable pulmonary adenocarcinoma lineage.
p40/p63/CK5/6	CAMS: negative/negative/negative. Earlier originating-hospital batch: focal/partial positivity.	negative/negative/negative	not reported	not reported	Institution- and batch-specific results are reported separately; they are not merged.
CD117	2+	3+	not reported	not reported	Descriptive only.
RB1	retained	retained	not reported	not reported	Atypical contextual finding; does not alone exclude SCLC.
p53 IHC	approximately 90% strong positive, missense-type pattern	approximately 95% strong positive, missense-type pattern	not reported	not reported	Keep analytically distinct from TP53 sequencing.
Ki-67	approximately 40% (CAMS consultation)	approximately 90% (CAMS consultation)	approximately 90% or greater	approximately 90% or greater	Reported separately by specimen, institution, and reviewed area; not averaged.
POU2F3	not reported in the CAMS consultation summary	not reported in the CAMS consultation summary	positive nuclear staining	positive nuclear staining	Subtype support only; percentage, intensity, and distribution were not reported.
GATA3/androgen receptor	not reported	not reported	negative/negative	negative/negative	Fudan review.
BRG1/SMARCA4/INI1	BRG1 retained/not reported	BRG1 retained/INI1 retained	not reported	not reported	SMARCA4-deficient tumor disfavored.
NUT	not reported	negative	not reported	not reported	NUT carcinoma disfavored.
PD-L1 SP263	not reported	TPS approximately 1%	not reported	not reported	Not used to infer response.
TP53 sequencing	not performed	TP53 p.Y236C; VAF 61.7%	not reported	not reported	Tissue sequencing of core; not lineage-specific.
ASCL1/NEUROD1/YAP1	not performed	not performed	not performed	not performed	Pretreatment material exhausted; no additional profiling.

CAMS, Chinese Academy of Medical Sciences Cancer Hospital. Fudan results are reported separately for the bronchoscopic and CT-guided core specimens. Values are not averaged across specimens or institutions.

### Diagnostic assessment

The principal diagnostic issue was lineage instability across two small-biopsy specimens. Both sites showed morphology favoring small cell carcinoma. Conventional neuroendocrine markers were absent, focal, or weak, and TTF-1 was negative. The early bronchoscopic batch showed focal squamous-marker staining, while RB1 expression was retained. The final interpretation was an integrated diagnosis most consistent with POU2F3-positive neuroendocrine-low SCLC, based on concordant morphology, specimen-specific immunophenotyping, and specialist review. POU2F3 did not establish the lineage diagnosis.

### Differential diagnosis

Poorly differentiated NSCLC was considered because the tumor was epithelial and TTF-1-negative, but neither stable glandular nor stable squamous differentiation was demonstrated in the two small biopsies. Basaloid squamous carcinoma remained a consideration because of focal squamous-marker staining in an earlier bronchoscopic batch; the formal bronchoscopic consultation and the core were p40-, p63-, and CK5/6-negative. POU2F3 positivity alone does not exclude basaloid squamous carcinoma (5, 6); the diagnosis depended on the overall morphologic and immunophenotypic context.

LCNEC remained in the differential because occasional core-biopsy cells had visible nucleoli and more cytoplasm. Given the limited pretreatment samples, a minor LCNEC component or an unsampled combined carcinoma could not be completely excluded; pCR left no viable untreated resection architecture for retrospective classification.

Other high-grade thoracic neoplasms were considered in this TTF-1-negative differential. SMARCA4-deficient undifferentiated thoracic tumor was disfavored by retained BRG1/SMARCA4 expression in both samples and retained INI1 in the core. NUT carcinoma was disfavored by negative NUT immunohistochemistry in the CT-guided core.

Retained RB1 expression reduced initial diagnostic confidence and prompted specialist review. RB1 loss is characteristic of conventional SCLC, but retained Rb protein has been documented in a minority of SCLC and cannot alone exclude the diagnosis (8). Retained Rb expression has also been reported more often in POU2F3-positive than POU2F3-negative resected SCLC in one cohort, but this association does not define an SCLC-P phenotype (9). In this case, RB1 was a contextual observation rather than a diagnostic determinant.

### Therapeutic intervention

Because treatment could not be indefinitely delayed while pathology review continued, the MDT used nab-paclitaxel, cisplatin, and pembrolizumab as an empiric bridge strategy under unresolved histologic classification. Four cycles of preoperative induction chemoimmunotherapy were administered. This course is not presented as a guideline-supported alternative to platinum-etoposide-based therapy for confirmed SCLC, for which established stage-adapted approaches remain the reference framework (10–13).

**Figure 2:**
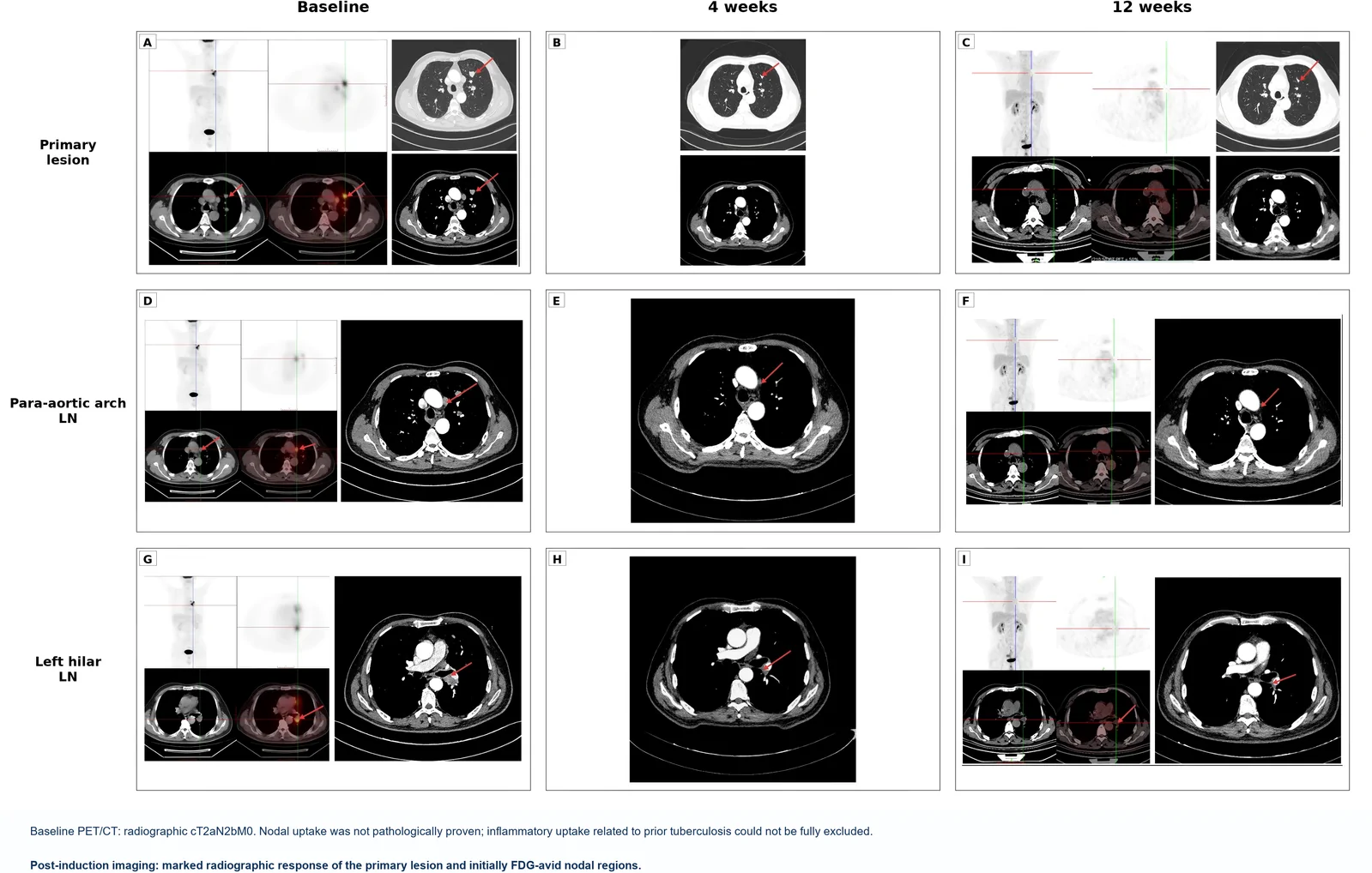
Imaging response. Serial PET/CT and CT images demonstrate the primary lesion **(A–C)**, para-aortic arch lymph node **(D–F)**, and left hilar lymph node **(G–I)** at baseline **(A, D, G)**, 4 weeks **(B, E, H)**, and 12 weeks **(C, F, I)**. Red arrows indicate the corresponding lesions or nodal regions. Post-induction imaging showed marked radiographic response of the primary lesion and initially FDG-avid nodal regions. The baseline cN2b designation was radiographic and not pathologically proven; inflammatory uptake related to previous tuberculosis could not be fully excluded.

After induction therapy, imaging showed marked radiographic response in the primary lesion and initially FDG-avid nodal regions (**Figure 2**). Repeated MDT review considered the deep response, the imaging-based baseline cN2b stage, the history of tuberculosis, and operative fitness. Surgery was selected as a highly individualized decision for pathologic assessment of residual primary and nodal disease, rather than as routine management for N2 SCLC (14–17).

### Follow-up and outcomes

**Figure 3:**
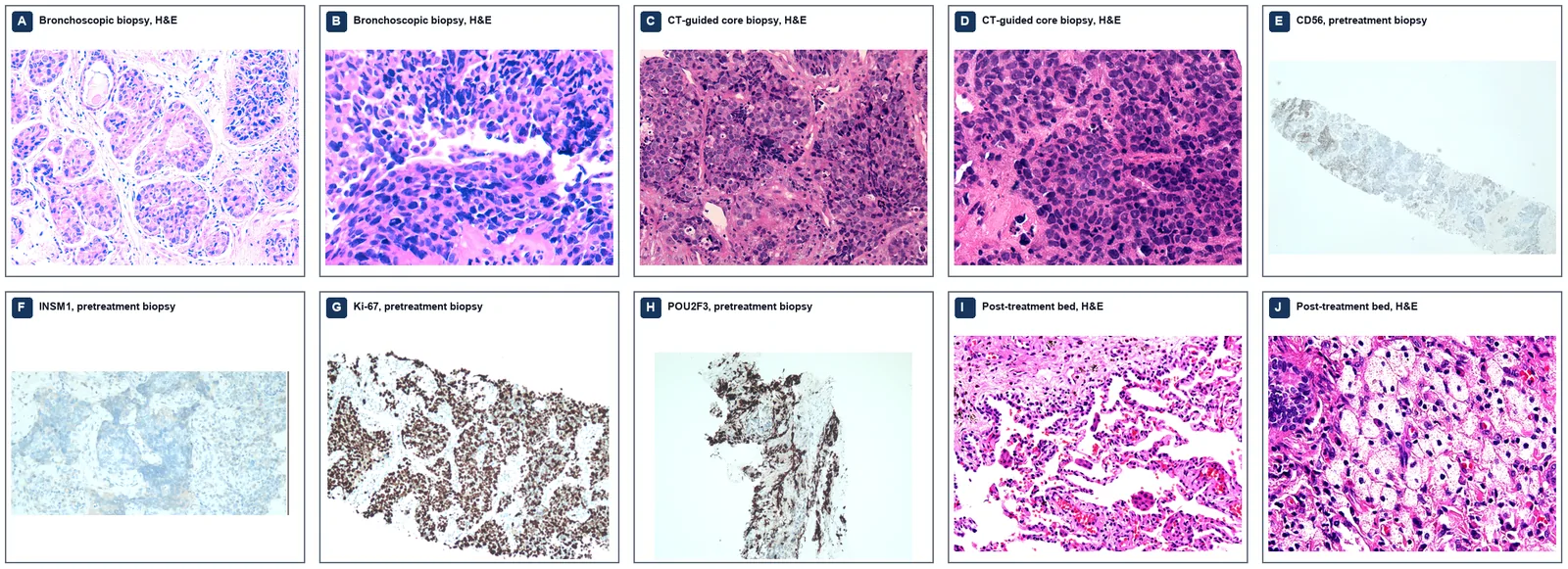
Pretreatment pathological findings and postoperative treatment response. **(A, B)** Representative H&E fields from the bronchoscopic biopsy obtained at the opening of the left-upper-lobe lingular bronchus, showing a high-grade carcinoma with small-cell cytological features. **(C, D)** Representative H&E fields from the anatomically distinct CT-guided core biopsy of the primary left-upper-lobe mass, showing a poorly differentiated high-grade tumor morphologically favoring small cell carcinoma. **(E)** CD56 immunostaining was positive in tumor cells. **(F)** INSM1 expression was negative or limited, illustrating the non-classical neuroendocrine immunophenotype. **(G)** Ki-67 immunostaining showed high proliferative activity. **(H)** POU2F3 nuclear staining was positive and supported subtype assignment within the integrated pathological diagnosis. **(I, J)** Postoperative H&E sections showed treatment-related fibrosis and histiocytic reaction without identifiable viable residual tumor.

The planned robot-assisted procedure was converted to open left upper lobectomy with systematic lymph node dissection. Postoperative pathology showed treatment-related changes without viable residual tumor in the primary bed and negative lymph nodes, consistent with pCR/ypT0N0M0 and 0% viable tumor cells. This endpoint clarified residual disease status but was not interpreted as evidence of cure. Representative pretreatment pathological findings and postoperative treatment-response findings are shown in **Figure 3**.

After multidisciplinary discussion, the patient completed four postoperative cycles of TP plus pembrolizumab. On May 26, 2026, bone scintigraphy, contrast-enhanced brain MRI, and contrast-enhanced chest and abdominal CT showed no evidence of recurrence or metastasis, and serum tumor markers remained within their respective reference ranges. Plasma MRD testing was performed using blood collected on June 10, 2026, and the report was issued on June 19, 2026. No ctDNA was detected, and the assay was interpreted as MRD-negative. Plasma hotspot variants were not detected. These findings are supportive surveillance information only and do not justify treatment de-escalation (18–23).

## Discussion

### Diagnostic instability in neuroendocrine-low POU2F3-positive SCLC

This case illustrates how neuroendocrine-low SCLC can remain lineage-ambiguous in limited material. The two pretreatment sites were anatomically distinct and both favored small cell morphology, yet the neuroendocrine and squamous-marker profiles were not uniform across specimen and institution. A specimen-aware approach preserved the actual discordances instead of averaging Ki-67 or merging discrepant squamous-marker results. The final diagnosis remained qualified because small biopsies cannot fully exclude minor LCNEC or an unsampled combined component. Normal ProGRP and NSE levels were biologically concordant with the neuroendocrine-low phenotype; however, these serum markers are neither sufficiently sensitive nor specific to establish SCLC or assign the POU2F3 subtype.

POU2F3 testing was clinically useful because morphology favored small cell carcinoma while the profile was TTF-1-negative, neuroendocrine-low, and complicated by retained RB1 and variable lineage markers. POU2F3 identified a biologically plausible tuft-cell-like subtype within an already integrated diagnosis. Its reported positivity lacked quantitative staining details, and ASCL1, NEUROD1, and YAP1 were not evaluated because material was exhausted. These limitations preclude a molecularly absolute assignment.

Lineage-associated transcriptional programs may extend beyond pulmonary SCLC. Metovic et al. identified distinct transcriptional profiles in extra-pulmonary neuroendocrine carcinomas, including differential expression of ASCL1, NEUROD1, POU2F3, YAP1, and other neuroendocrine-lineage regulators (24). These observations suggest that transcription factor-based classification may reflect broader lineage biology across high-grade neuroendocrine carcinomas. In the present case, POU2F3 was interpreted with morphology, conventional immunohistochemistry, and clinicopathological correlation to support subtype assignment rather than as a stand-alone diagnostic determinant.

Accordingly, the small-biopsy differential includes poorly differentiated NSCLC, basaloid squamous carcinoma, LCNEC or combined carcinoma, and other high-grade thoracic neoplasms. Their distinction requires morphology, specimen-specific panel-level immunohistochemistry, and clinicoradiological correlation rather than POU2F3 in isolation.

### Bridge treatment under unresolved histology

For a clinically time-sensitive high-grade thoracic malignancy, management may begin before every diagnostic question is closed. In this patient, TP plus pembrolizumab was an empiric bridge strategy under unresolved histologic classification and was repeatedly reassessed as pathology became more stable. The response does not establish superiority, equivalence, or an alternative standard for confirmed SCLC. This distinction is essential because a favorable individual response can otherwise be mistaken for evidence of a general treatment strategy.

### Therapeutic, immune, and prognostic implications

Potential therapeutic and genomic implications. POU2F3-positive SCLC currently has no established subtype-specific targeted therapy. Preclinical studies have identified IGF1R dependence in POU2F3-high models and implicated mammalian SWI/SNF complex activity in maintaining the POU2F3 transcriptional program (3, 25). These findings remain investigational and did not guide treatment in the present case.

PTEN inactivation and MYC amplification have been reported in subsets of POU2F3-positive SCLC (5). In this patient, no PTEN alteration was detected within the genomic regions and alteration types covered by the 41-gene targeted panel. However, because this was a hotspot-based assay rather than comprehensive whole-gene profiling, PTEN inactivation outside the assay scope could not be completely excluded. MYC was not included in that panel; a separate extended tissue-profiling report identified MYC copy-number gain. The original copy-number gain terminology was retained and was not reclassified as definitive amplification. These findings were descriptive, nonactionable, and were not interpreted as explanations for the pCR.

The high Ki-67 index may be compatible with chemotherapy responsiveness but is insufficient to explain the depth of response. Low PD-L1 expression, low tumor mutational burden, and microsatellite stability likewise provide no clear explanation. The respective contributions of chemotherapy, pembrolizumab, and individual tumor biology cannot be separated.

Immune context. Neuroendocrine-low SCLC has been associated with increased immune-cell infiltration, sometimes described as an immune-oasis phenotype (26, 27). However, this feature could not be reliably assessed in the present case because the pretreatment biopsies were limited and no dedicated CD3, CD8, or CD68 immune-cell quantification, spatial profiling, or multiplex immunofluorescence was performed. The postoperative specimen showed pCR; treatment-related inflammatory and histiocytic changes therefore could not be assumed to represent the pretreatment immune microenvironment.

Prognostic context. Reported prognostic associations of POU2F3-positive SCLC remain inconsistent. Some resected cohorts have suggested more favorable outcomes in POU2F3-high tumors, whereas other cohorts have shown no consistent overall-survival advantage or have associated SCLC-P with poorer outcome (5, 28, 29). Differences in cohort size, stage distribution, treatment, surgical-case proportion, subtype-assignment method, and transcriptomic versus immunohistochemical definitions may contribute. POU2F3 positivity alone is not an established favorable or adverse prognostic biomarker. The short follow-up, pCR, negative imaging, normal tumor markers, and MRD-negative plasma result do not establish long-term prognosis or cure.

## Conclusion

Neuroendocrine-low POU2F3-positive SCLC can present as a diagnostically unstable high-grade thoracic malignancy. Morphology, specimen-specific immunophenotyping, and specialist review should establish the diagnosis, with POU2F3 used to support subtype assignment. MDT reassessment may guide individualized management when histology remains unresolved, and surgery can provide pathologic assessment in highly selected circumstances. This single case does not define standard treatment or long-term prognosis.

## Data Availability

The original clinical, imaging, pathological, MRD, and genomic reports contain potentially identifying patient information and are not publicly available to protect patient privacy. De-identified data supporting the conclusions of this article are included in the article. Further reasonable inquiries may be directed to the corresponding author, subject to institutional and ethical restrictions.
