# Supplementary material for: Case Report: Neuroendocrine-low POU2F3-positive small cell lung cancer presenting as a diagnostically unstable thoracic malignancy
Source: Front Oncol. 2026 Sep 4;16:1917451. doi: 10.3389/fonc.2026.1917451 (PMC13585606; doi:10.3389/fonc.2026.1917451)
Supplement: Supplementary file 1 [file Table1.docx]

**CARE Checklist**

*Case Report: Neuroendocrine-low POU2F3-positive small cell lung cancer presenting as a diagnostically unstable thoracic malignancy*

*Completed for supplementary submission. All page and continuous-line references were verified against the final 12-page clean/accepted-text manuscript; tracked-manuscript pagination was not used.*

| **CARE item** | **Checklist detail** | **Manuscript location** | **Status** |
| --- | --- | --- | --- |
| **Title** | Diagnosis or intervention is identified in the title. | Page 1, lines 1-2 | Reported |
| **Keywords** | Key words identify the case topic. | Page 1, lines 22-23 | Reported |
| **Abstract** | Background, case presentation, and conclusion are summarized. | Page 1, lines 3-21 | Reported |
| **Introduction** | Medical context and rationale are provided. | Pages 1-2, lines 24-38 | Reported |
| **Patient information** | Demographics, relevant history, symptoms, and family history. | Page 2, lines 40-46 | Reported |
| **Clinical findings** | Baseline imaging and radiographic staging findings. | Page 2, lines 47-52 | Reported |
| **Timeline** | Chronology of diagnostic and treatment events. | Figure 1 legend, pages 11-12, lines 344-353; Figure 1 submitted separately. | Reported |
| **Diagnostic assessment** | Pathology, differential diagnosis, limitations, and molecular context. | Pages 2-5, lines 53-123; Table 1, page 4, lines 91-93 | Reported |
| **Therapeutic intervention** | Four cycles of induction chemoimmunotherapy, surgery, and four cycles of postoperative systemic therapy with TP plus pembrolizumab. | Pages 5-6, lines 124-135 and 141-142; Figure 1 legend, pages 11-12, lines 344-353 | Reported |
| **Follow-up and outcomes** | pCR/ypT0N0M0, May 26 imaging and tumor markers, June 10 MRD blood collection, and June 19 MRD report. | Page 6, lines 136-147 | Reported |
| **Discussion** | Strengths, limitations, and cautious interpretation. | Pages 6-8, lines 148-212 | Reported |
| **Patient perspective** | Patient perspective was not available for inclusion in this report. | Not available | Not available |
| **Informed consent** | Written informed consent for case details and images. | Page 8, lines 221-224 | Reported |
